# 

**Published:** 1900-06

**Authors:** 


### CONTENTS.

ORIGINAL COMMUNICATIONS. PAGE

New Removable Facing. By CEPHAS WHITNEY, D.D.S., Jamaica, W. I. . . . . 373
Longevity of Dentists. By WALDO E. BOARDMAN, D.M.D., Boston, Mass. . . 376
Alloys for Filling Purposes. By A. P. FELLOWS, D.D.S., Philadelphia . . . . 379
Some Effects of Immigration from a Dental View. By FRANK R. DICKERMAN, D.M.D., Taunton, Mass. . . . . . . . . . . . . . . . . . . . 384
The Newness of Dentistry. By CHARLES W. ELIOT, LL.D. . . . . . . . . 389
The Union of Dental and Medical School. By DR. C. S. MINOT, Boston, Mass. . 393
The Hand the Servant of the Brain. By DR. E. A. BOGUE, New York . . . . 397
Dental Service as a Ministry to Life. By REV. DR. GEORGE A. GORDON . . . . 402
Dentistry in its Relation to Medicine. By DR. GEORGE S. ALLAN, New York . . 406

REPORTS OF SOCIETY MEETINGS.

Academy of Stomatology . . . . . . . . . . . . . . . . . . . . 408
Union Meeting of The New York Institute of Stomatology and the American Academy of Dental Science . . . . . . . . . . . . . . . . . . 417
Harvard Odontological Society . . . . . . . . . . . . . . . . . 418

EDITORIAL.

The Duties and Responsibilities of the Editor . . . . . . . . . . . 426
The Death of Dr. Cushing . . . . . . . . . . . . . . . . . . . 429

DOMESTIC CORRESPONDENCE.

On the Use of Nitrate of Silver . . . . . . . . . . . . . . . . 430

NOTES AND COMMENTS . . . . . . . . . . . . . . . . . . . . 430

CURRENT NEWS.

Resolutions regarding Dr. J. N. Crouse and the Dental Protective Association . . 432
National Dental Association, Section III. . . . . . . . . . . . . . 433
Massachusetts Dental Society . . . . . . . . . . . . . . . . . . 434
International Dental Congress . . . . . . . . . . . . . . . . . . 434
Wisconsin State Dental Society . . . . . . . . . . . . . . . . . 436
California State Dental Association . . . . . . . . . . . . . . . 436
Central Dental Association of Northern New Jersey . . . . . . . . . 436
Colorado State Dental Association . . . . . . . . . . . . . . . . 436

